# Prolonged Acetylsalicylic-Acid-Supplementation-Induced Gastritis Affects the Chemical Coding of the Stomach Innervating Vagal Efferent Neurons in the Porcine Dorsal Motor Vagal Nucleus (DMX)

**DOI:** 10.1007/s12031-014-0274-y

**Published:** 2014-03-19

**Authors:** Marta Gańko, Jarosław Całka

**Affiliations:** Department of Clinical Physiology, Faculty of Veterinary Medicine, University of Warmia and Mazury in Olsztyn, Oczapowskiego 13, 10-718 Olsztyn, Poland

**Keywords:** Acetylsalicylic acid, PACAP, VIP, NOS, GAL, Pig

## Abstract

The main goal of our research was to study the possible alterations of the chemical coding of the dorsal motor vagal nucleus (DMX) neurons projecting to the porcine stomach prepyloric region following prolonged acetylsalicylic acid supplementation. Fast Blue (FB) was injected into the studied area of the stomach. Since the seventh day following the FB injection, acetylsalicylic acid (ASA) was given orally to the experimental gilts. All animals were euthanized on the 28th day after FB injection. Medulla oblongata sections were then processed for double-labeling immunofluorescence for choline acetyltransferase (ChAT), pituitary adenylate cyclase-activating peptide (PACAP), vasoactive intestinal polypeptide (VIP), nitric oxide synthase (NOS), galanin (GAL), substance P (SP), leu enkephalin (LENK), and cocaine- and amphetamine-regulated transcript (CART). In the control DMX, only PACAP was observed in 30.08 ± 1.97 % of the FB-positive neurons, while VIP, NOS, GAL, SP, LENK, and CART were found exclusively in neuronal processes running between FB-labeled perikarya. In the ASA DMX, PACAP was revealed in 49.53 ± 5.73 % of traced vagal perikarya. Moreover, we found de novo expression of VIP in 40.32 ± 7.84 %, NOS in 25.02 ± 6.08 %, and GAL in 3.37 ± 0.85 % of the FB-labeled neurons. Our results suggest that neuronal PACAP, VIP, NOS, and GAL are mediators of neural response to aspirin-induced stomach inflammatory state.

## Introduction

Acetylsalicylic acid (ASA, aspirin) is a nonsteroidal anti-inflammatory drug (NSAID) which has been commonly used to relieve pain and fever. Due to its antiplatelet action, aspirin is often applied for cardiovascular protection (Hsu 2012). Although utilized in human medicine for centuries, its mechanism of action was only uncovered in the second half of the 20th century (Vane 1971).

ASA operates through inhibition of cyclooxygenase (COX). COX allows the synthesis of tromboxane A_2_, prostacyclins, and prostaglandins from arachidonic acid. According to available reports, COX occurs in three isoforms: COX-1, COX-2, and COX-3. COX-1 is constitutively expressed in the majority of cells and in the stomach; it is directly responsible for gastric mucosa protection. COX-2 is mainly induced by inflammatory processes, and COX-3 is a variant of COX-1 found in the nervous system (Vane and Botting 2003; Fuster and Sweeny 2011). Aspirin completely and irreversibly inactivates COX-1 and also affects COX-2, because of its 60 % compliance with COX-1. Therefore, ASA is used as an antiplatelet agent and as a drug for the treatment of inflammatory processes. However, for the same reason, aspirin is responsible for a wide range of undesirable actions.

It was found that even a single dose of aspirin resulted in slight superficial damage to the gastric mucosa cells, which is often quickly repaired by natural healing mechanisms. This problem becomes much more serious when the regeneration processes are disturbed and erosions in gastric mucosa, mainly in prepyloric area and antrum, may then occur (Yeomans 2011). Moreover, aspirin-induced inhibition of COX-1 resulted in interruption of prostaglandin synthesis. Consequently, the gastric mucosa becomes unprotected against injury, and a reduction of mucosal bicarbonate and mucus secretion and decrease of local blood flow take place (Cryer 1999; Shiotani et al. 2008).

The dorsal motor nucleus of the vagus nerve provides extrinsic efferent parasympathetic innervation of the stomach (Yoshida et al. 1989; Okumura and Namiki 1990; Siaud et al. 1990; Gańko and Całka 2013) which is responsible for the regulation of gastric motility, blood flow, secretion, gastric mucosal protection, and ulcer formation (Tache and Yoneda 1993; Travagli et al. 2003; Zhou et al. 2008). Our previous research proved that peripheral transection of the vagal efferent processes at the level of gastric wall evoked induction of the pituitary adenylate cyclase-activating peptide (PACAP), vasoactive intestinal polypeptide (VIP), nitric oxide synthase (NOS), and galanin (GAL) expression in response to a traumatic neuronal injury of the stomach supplying DMX perikarya (Gańko and Całka 2013). This phenomenon has been also reported in the porcine descending colon (Gonkowski et al. 2010; Gonkowski and Całka 2012) where both axotomy and an inflammatory state stimulated expression of PACAP and GAL in the intestinal ganglia. Previous reports underline possible neuroprotective and/or neurotrophic function of PACAP, VIP, GAL, and NO (Gomariz et al. 2001; Somogyvari-Vigh and Reglodi 2004; Suarez et al. 2006; Elliott-Hunt et al. 2011; Shioda and Gozes 2011; Zhihui 2013).

Moreover, our former data (Gańko and Całka 2013) showed characteristic distribution pattern of substance P (SP), leu enkephalin (LENK), and cocaine- and amphetamine-regulated transcript (CART) immunoreactive (IR) fibers in the DMX, indicating involvement of those neurotransmitters/neuromodulators in modulation of various gastric functions mediated by vagus nerve (Spencer and Talman 1986; Yang and Tache 1997; Browning et al. 2002; Okumura et al. 2000; Smedh and Moran 2006). Some reports also pointed to the participation of SP, LENK, and CART in processes related to gastritis as well as neurogenic or chemically induced inflammation (Gyires et al. 2001; Sipos et al. 2006; Burliński 2012).

Although gastritis constitutes a common complication of aspirin treatment, there is a complete lack of knowledge concerning the chemical coding of the gastric vagal efferent neurons following ASA-induced stomach inflammation. Therefore, the main purpose of our study was to investigate possible alterations of chemical coding of the vagal efferent perikarya supplying the stomach prepyloric region following aspirin-induced gastritis in pigs, a widely-used animal model for human biomedical research (Verma et al. 2011; Swindle et al. 2012).

## Materials and Methods

### Animals and Experimental Procedures

This experiment was carried out on ten immature gilts of the Large White Polish breed (about 20 kg of body weight). Animals were housed in standard laboratory conditions with access to tap water and species-specific chow. The experimental procedures applied have been in accordance with EU Directive 2010/63/EU and were approved by the local ethics committee in Olsztyn (decision no. 05/2010).

All animals were subjected to general anesthesia induced by azaperone (Stresnil, Jansen Pharmaceutica N.V., Belgium; 4 mg/ kg of body weight, i.m.) and sodium thiopental (Thiopental, Sandoz, Kundl-Rakusko, Austria; 10 mg/kg of body weight, i.v.). A gastroscopic examination was then applied in order to confirm the physiological state of the gastric mucosa. Median laparotomy was performed to investigate the localization of the parasympathetic DMX neurons which innervate the gastric prepyloric area. Following stomach exposure, a total volume of 50 μl of a 5 % aqueous suspension of the fluorescence retrograde neuronal tracer Fast Blue (FB, EMS-CHEMIE GmbH, Germany) was injected into the studied region of all animals. The diamond-shaped part of the prepyloric region of the anterior stomach wall (ca. 4 cm × 4 cm) was injected several times (*n* = 50, 1 μl per one injection) with FB using a Hamilton syringe equipped with a 26-gauge needle. In order to avoid the diffusion of the tracer into surrounding tissues, the needle was left in place for about 20 s after each injection. The gilts were then allocated randomly to a control group (*n* = 5) and the ASA-treated group (ASA, *n* = 5).

From the seventh day after the FB injection, the gilts of the ASA group were treated with ASA (aspirin, BAYER; 100 mg/kg b.w.) given orally everyday, 1 h before feeding, until the 27th day of the experiment. On the 28th day, all animals were deeply re-anesthetized as depicted above and underwent gastroscopic examination, which confirmed the presence of gastritis symptoms in gilts of the ASA group. Directly following gastroscopy, all (control and ASA) animals were euthanized by an overdose of sodium thiopental and then perfused transcardially with 4 % buffered paraformaldehyde (pH 7.4). For histopathological examination, fragments of the wall of the prepyloric stomach area were collected from gilts of the ASA group to confirm pathological microscopic changes in this organ.

### Immunohistochemistry

Medulla oblongata blocks were collected from all the studied animals and postfixed in the same fixative for 20 min, rinsed in 0.1 M PB (pH 7.4) over 3 days, finally transferred to a 30 % buffered sucrose solution (pH 7.4) containing 0.01 % natrium azide, and stored at 4 °C.

Fourteen-micrometer-thick serial cryostat sections were prepared and mounted on chrome alum-coated slides and then evaluated under an Olympus BX51 fluorescent microscope equipped with an appropriate filter for FB to localize and count the FB-labeled neurons. Only cell bodies with visible nucleus, in every fourth section, were scored to avoid their double counting.

Selected slides containing FB-positive perikarya were processed for the immunocytochemical staining procedure. Briefly, after air-drying at room temperature for 45 min, the sections were washed 3 × 10 min in a 0.1 M phosphate-buffered saline (PBS, pH 7.4), incubated for 1 h in a blocking buffer containing: 0.1 % bovine serum albumin (BSA) in 0.1 M PBS, 1 % Triton X-100, 0.05 % Thimerosal, and 0.01 % sodium azide, again rinsed in PBS (3 × 10 min), and then incubated overnight at room temperature with a mixture of primary antibodies (Table 1).

**Table 1:** Description of antibodies

Antigen	Species	Code	Dilution	Manufacturer/supplier
Primary antibodies				
CART	Rabbit	H-003-61	1:10,000	Phoenix Pharmaceuticals, USA
ChAT	Goat	AB144P-1ML	1:50	Millipore, USA
GAL	Rabbit	AB2233	1:5,000	Millipore, USA
LENK	Mouse	4140-0355	1:500	AbD Serotec, UK
NOS	Rabbit	AB5380	1:8,000	Chemicon, USA
PACAP	Guinea pig	T-5039	1:300	Bachem, USA
SP	Rat	450-0505	1:300	AbD Serotec, UK
VIP	Rabbit	PEPA41T	1:6,000	AbD Serotec, UK
Secondary antibodies				
Alexa Fluor 488 nm anti-goat		A11055	1:1,000	Invitrogen, USA
Alexa Fluor 546 nm anti-guinea pig		A11074	1:1,000	
Alexa Fluor 546 nm anti-mouse		A10036	1:1,000	
Alexa Fluor 546 nm anti-rabbit		A10040	1:1,000	
Alexa Fluor 546 nm anti-rat		A11081	1:1,000	

Following subsequent washing in PBS (3 × 10 min), the sections were incubated at room temperature for 1 h with appropriate secondary antibodies (Table 1) and again rinsed in PBS (3 × 10 min). After staining, the slides were cover-slipped with carbonate-buffered glycerol (pH 8.6).

For omission control purposes, the primary antibodies were omitted from the applied staining protocol, while for replacement control purposes, the sections were stained with normal sera instead of primary antibodies. Staining was not observed in either case.

The sections were then evaluated under an Olympus BX51 microscope equipped with epifluorescence and appropriate filter sets and then photographed.

### Statistical Analysis

The obtained data were processed statistically using Statistica 10 software (StatSoft Inc., Tulsa, OK, USA) and expressed as a mean ± standard error of mean (SEM). The significance of differences was estimated using the Student’s *t* test for independent samples. Differences with a probability of *P* < 0.05 were considered significant or highly significant when *P* < 0.001.

## Results

**Fig. 1:**
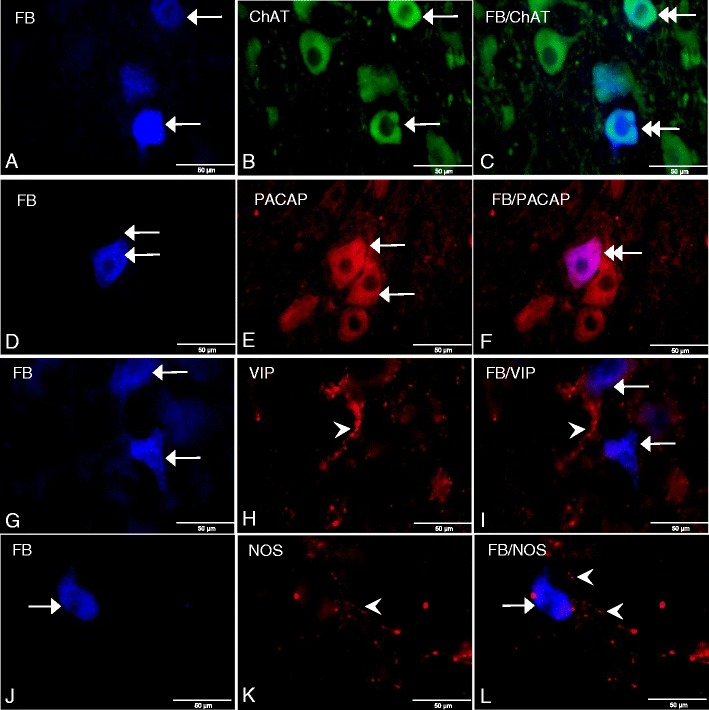
**a** FB-labeled neurons (*arrows*) in the porcine control DMX. **b** ChAT-IR neurons (*arrows*) in the DMX. **c** Double-labeled FB/ChAT-IR neurons (*double arrows*) in the DMX. **d** FB-labeled neuron (*arrow*) in the porcine control DMX. **e** PACAP-IR neurons (*arrows*). **f** Double-labeled FB/PACAP-IR neuron (*double arrow*). **g** FB-labeled neurons (*arrows*) in the porcine control DMX. **h** VIP-IR fiber (*arrowhead*) in the DMX. **i** VIP-IR fiber (*arrowhead*) in close neighborhood of the FB-labeled somata (*arrows*) in the DMX. **j** FB-labeled neuron (*arrow*) in the porcine control DMX. **k** NOS-IR fibers (*arrowheads*) in the DMX. **l** NOS-IR fibers (*arrowheads*) in close proximity of the FB-labeled perikaryon (*arrow*) in the DMX. Photographs of Fig. 1c, f, i, l were prepared by superimposition of single images

As we reported recently, the FB-labeled perikarya were observed to be scattered throughout the whole extent of both left and right DMX (Gańko and Całka 2013). Although the highest concentration of the FB-positive cells was detected in the middle nuclear region, their number decreased gradually toward the rostral and caudal pools of DMX where only single-labeled somata were found. The majority of the FB-positive neurons were oval, round, or multipolar in shape with a centrally situated nucleus and measured about 20 to 50 μm in diameter. The intensity of the FB-fluorescence in labeled cell bodies varied from intense to weak, independent of their location in the nucleus. Moreover, microscopic analysis showed that all FB-labeled perikarya expressed specific cholinergic neuronal marker ChAT confirming their parasympathetic character (Fig. 1a, b, c).

### Control Group

Gastroscopic examination confirmed the physiological state of the stomach mucosa membrane.

Microscopic analysis revealed 275 ± 22.63 FB-positive cell bodies per animal in the left side DMX, while 210 ± 26.9 perikarya were found in the right DMX. The total number of FB-labeled neurons amounted to 485.2 ± 42.7 perikarya per animal.

Immunostaining with anti-PACAP antibodies detected PACAP expression in 30.08 ± 1.97 % of the FB-labeled neurons (Fig. 1d, e, f), whereas VIP, NOS, GAL, SP, LENK, and CART immunoreactivities were found exclusively in nerve fibers running in close proximity to the DMX perikarya.

**Fig. 2:**
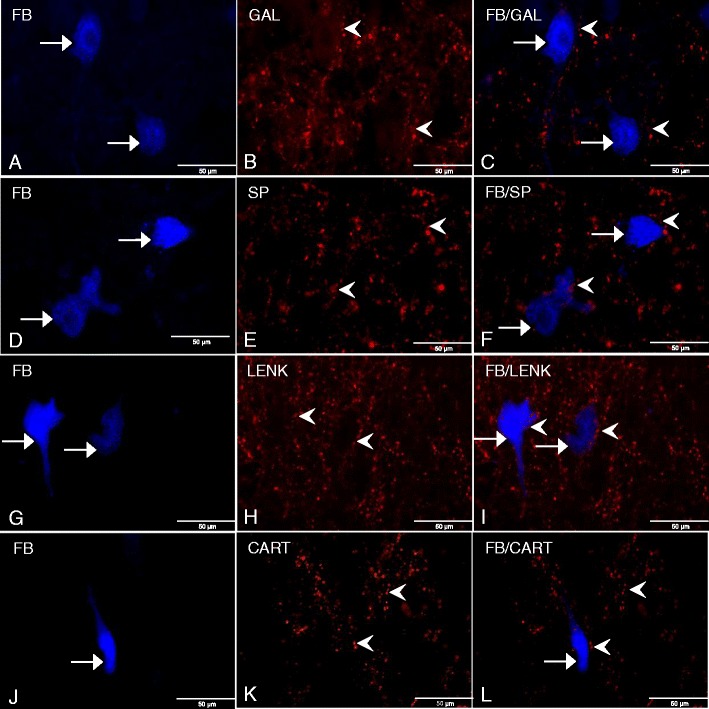
**a** FB-labeled neurons (*arrows*) in the porcine control DMX. **b** GAL-IR fibers (*arrowheads*) in the DMX. **c** GAL-IR fibers (*arrowheads*) in close proximity of the FB-labeled somata (*arrows*) in the DMX. **d** FB-labeled neurons (*arrows*) in the porcine control DMX. **e** SP-IR fibers (*arrowheads*) in the DMX. **f** SP-IR fibers (*arrowheads*) surrounded the FB-labeled somata (*arrows*) enabling direct contact. **g** FB-labeled neurons (*arrows*) in the porcine control DMX. **h** LENK-IR fibers (*arrowheads*) in the DMX. **i** LENK-IR fibers (*arrowheads*) formed basket-like structures encircling FB-labeled neurons (*arrows*) in the DMX. **j** FB-labeled neuron (*arrow*) in the porcine control DMX. **k** CART-IR fibers (*arrowheads*) in the DMX. **l** CART-IR fibers (*arrowheads*) in close proximity of the FB-positive perikaryon (*arrow*) in the DMX. Photographs of Fig. 2c, f, i, l were prepared by superimposition of single images

Although PACAP-IR/FB-positive somata were identified throughout the entire area of both the left and right DMX, they did not create any characteristic distribution pattern. Only the single PACAP immunoreactive neuronal fibers were rarely observed in the nuclear matrix. Immunostaining revealed the presence of single, fine VIP-IR processes which sometimes ran near the FB-positive perikarya (Fig. 1g, h, i). Microscopic examination also revealed varicose NOS-IR fibers running rarely in close proximity to the FB-labeled neurons (Fig. 1j, k, l). Moreover, moderately dense GAL-IR fibers dispersed throughout the studied area were observed running near the FB-labeled cell bodies (Fig. 2a, b, c). The dense network of the SP immunoreactive varicose processes often formed basket-like structures tightly surrounding the FB-labeled perikarya (Fig. 2d, e, f). Likely, pronounced LENK-IR processes encircled the FB-labeled gastric neurons, forming basket-like structures (Fig. 2g, h, i).

Finally, fine CART-IR fibers penetrating the intercellular matrix and occasionally surrounding FB-positive somata were also noted (Fig. 2j, k, l).

### ASA Group

**Fig. 3:**
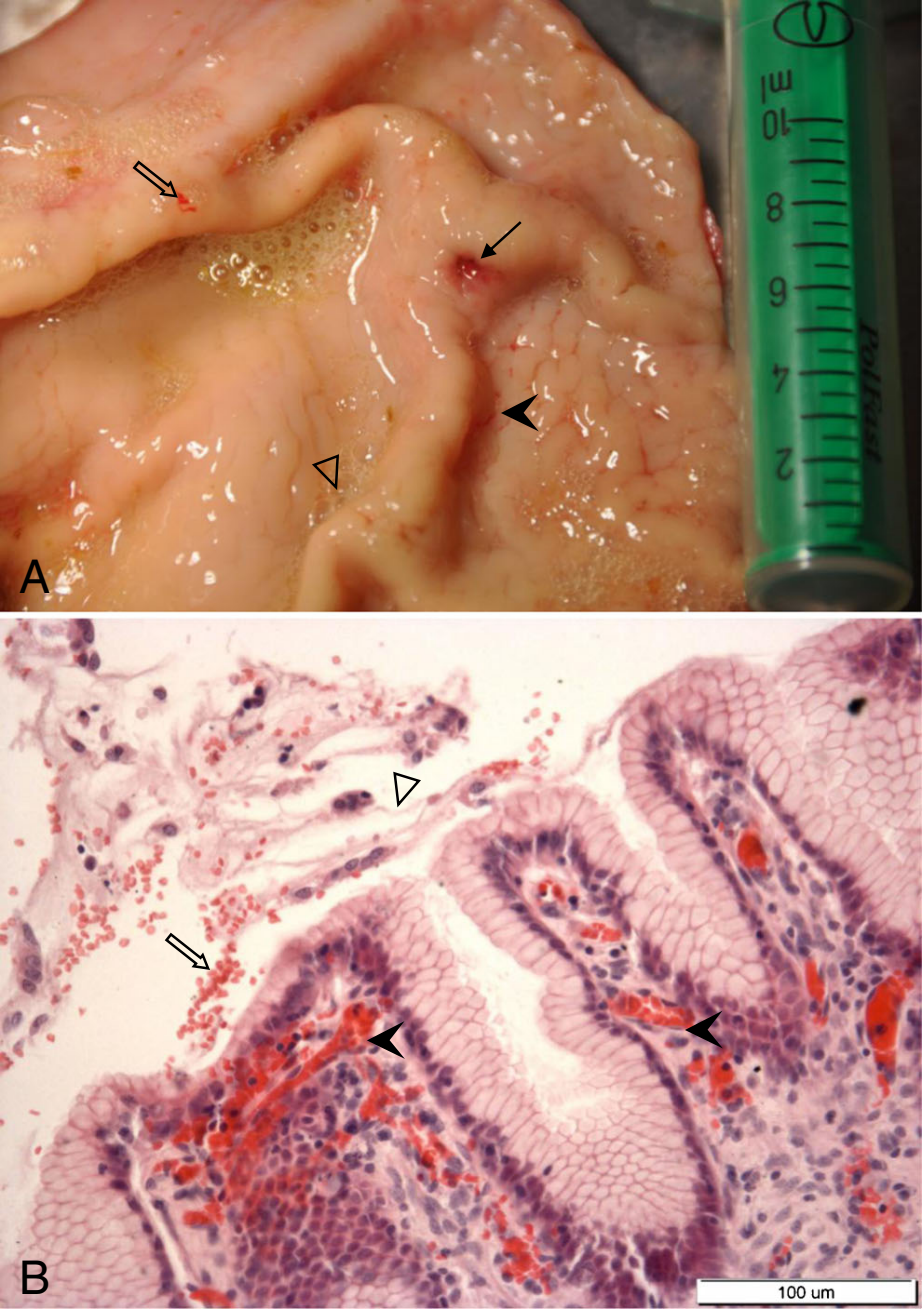
**a** Inflammatory changes in the gastric mucosa membrane following acetylsalicylic acid supplementation: superficial lesion of gastric mucosa (*full arrow*), hyperemia (*full arrowhead*), excessive mucus (*empty arrowhead*), and blood in the lumen (*empty arrow*). **b** Hyperemia (*full arrowheads*) in gastric mucosa, red blood cells (*empty arrow*), and excessive amount of mucus (*empty arrowhead*) in stomach lumen

A gastroscopic examination performed on the 28th day of the experiment revealed macroscopic changes in the gastric mucosa, such as hyperemia, petechiae in the gastric mucosa, the presence of small ulcers and superficial lesions, fragility of gastric and duodenum mucosa, as well as inflammatory changes in the duodenum. These symptoms were confirmed during a postmortem anatomopathological examination (Fig. 3a).

**Fig. 4:**
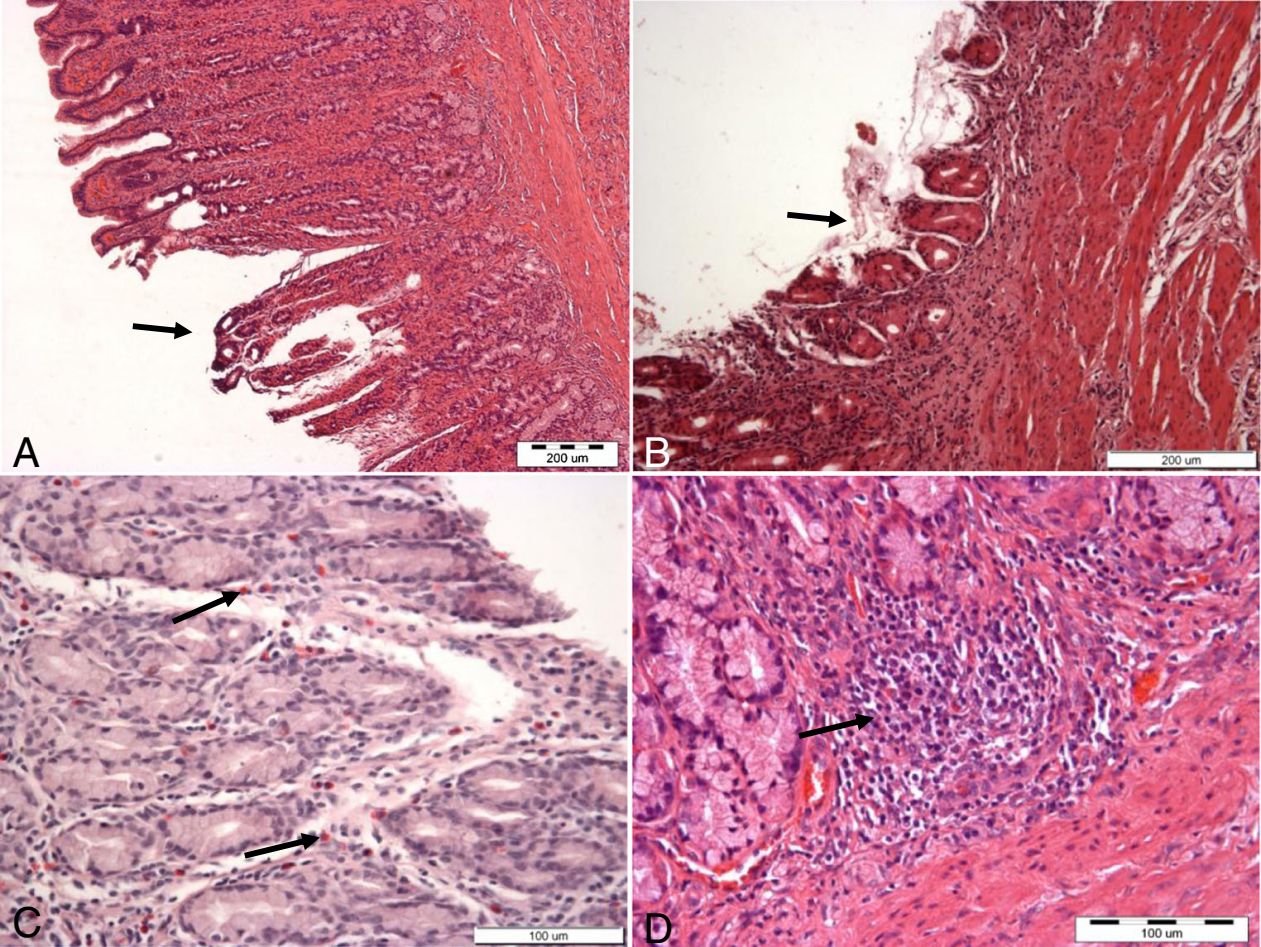
**a** Superficial erosion (*arrow*) in gastric mucosa. **b** Deep erosion (*arrow*) in gastric mucosa. **c** Presence of eosinophilic cells (*arrows*) in gastric mucosa. **d** Proliferation of lymphatic cells (*arrow*) in gastric mucosa

Histopathological examination of fragments of the wall of the gastric prepyloric region confirmed pathological microscopic changes, such as superficial and deep erosions in the gastric mucosa, hyperemia, foliculosis, proliferation of the lymphocytes, and infiltration of eosinophilic cells. Moreover, excessive mucus and red blood cells were observed in the stomach lumen (Figs. 3b and 4a, b, c, d).

We found 705.8 ± 61.04 of FB-labeled perikarya in the ASA DMX. A total of 389.8 ± 29.65 of the FB-labeled neurons were detected in the left side DMX, while right side nucleus contained 316 ± 31.91 somata. Their morphology and distribution pattern resembled that observed in the control group.

**Fig. 5:**
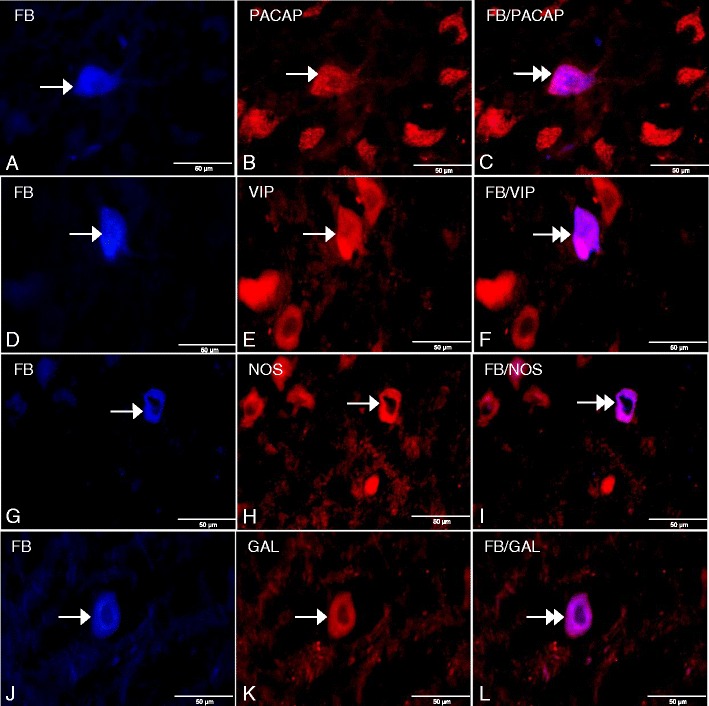
**a** FB-labeled neuron (*arrow*) in the ASA DMX. **b** PACAP-IR neuron (*arrow*) in the DMX. **c** Double-labeled FB/PACAP-IR neuron (*double arrow*) in the DMX. **d** FB-labeled neuron (*arrow*) in the ASA DMX. **e** VIP-IR neuron (*arrow*) in the DMX. **f** Double-labeled FB/VIP-IR perikaryon (*double arrow*) in the DMX. **g** FB-labeled neuron (*arrow*) in the ASA DMX. **h** NOS-IR neuron (*arrow*) in the DMX. **i** Double-labeled FB/NOS-IR perikaryon (*double arrow*) in the DMX. **j** FB-labeled neuron (*arrow*) in the ASA DMX. **k** GAL-IR neuron (*arrow*) in the DMX. **l** Double-labeled FB/GAL-IR perikaryon (*double arrow*) in DMX. Photographs of Fig. 5c, f, i, l were prepared by superimposition of single images

Prolonged ASA supplementation altered the chemical phenotype of the FB-positive cell bodies (Table 2). Examination of the sections stained with antibody against PACAP showed that 49.53 ± 5.73 % of retrogradely traced gastric neurons expressed PACAP immunoreactivity (Fig. 5a, b, c). Approximately 40.32 ± 7.84 % of FB-positive perikarya stained for VIP immunoreactivity (Fig. 5d, e, f), while the occurrence of this peptide in control neurons was not detected. Analysis of sections incubated with anti-NOS antibody showed that 25.02 ± 6.08 % of the FB-traced neurons exhibited NOS immunoreactivity (Fig. 5g, h, i), whereas in the control group, all FB-positive somata were devoid of NOS immunofluorescence. Moreover, immunolabeling with anti-GAL antibody revealed that 3.37 ± 0.85 % of the FB-positive perikarya demonstrated GAL immunoreactivity (Fig. 5j, k, l), while in the control animals, no GAL-IR/FB-labeled somata were found.

**Table 2:** Average percentage of the FB-labeled neurons containing PACAP, VIP, NOS, and GAL

Antigen	Control (%)	ASA (%)
PACAP	30.08 ± 1.97	49.53 ± 5.73*
VIP	0	40.32 ± 7.84**
NOS	0	25.02 ± 6.08*
GAL	0	3.37 ± 0.85*

Data are shown as the mean ± standard error of mean (SEM). The significance of differences was estimated using the Student’s *t* test for independent samples

*P < 0.05; **P < 0.001

In general, all FB-positive neurons expressing PACAP, VIP, NOS, and GAL were irregularly scattered throughout the entire area of the studied nucleus.

Despite the presence of PACAP, VIP, NOS, and GAL immunoreactivities in perikarya, the distribution and spatial organization of neuronal fibers containing PACAP, VIP, NOS, GAL, SP, LENK, and CART in the ASA group were similar to those in the control group.

## Discussion

This study provides data on adaptive alterations of chemical coding of the DMX neurons projecting to the porcine stomach prepyloric area (referred further as gastric neurons), following long-term ASA supplementation.

We found that in control animals, approximately 30 % of the FB-positive neurons expressed PACAP, whereas aspirin-evoked gastritis induced PACAP expression in nearly 50 % of the gastric neurons. Our discovery implicates that PACAP-IR vagal perikarya are a key player in the stomach inflammatory response to ASA-generated pathology and is congruent with a previously reported increase of PACAP-IR nerve fibers in the enteric nervous system of porcine descending colon as a consequence of various inflammatory factors (Gonkowski and Całka 2012). Indeed, PACAP inhibited the production of pro-inflammatory cytokines (interleukin (IL)-1β, IL-6, IL-12) in the colon (Azuma et al. 2008). Moreover, increased expression of PACAP and its receptors after injury may point to a possible modulating effect of PACAP in inflammatory process (Delgado et al. 1999; Waschek 2013). Available reports suggest that in addition to neuronal PACAP, some immunocompetent cells may constitute an additional nonneuronal source (Abad et al. 2002), in consequence, strengthening the position of the neuropeptide in peripheral immune response. PACAP also has been found to promote neuronal survival, neuronal protection (Brenneman 2007; Bourgault et al. 2011; Tamas et al. 2012), and axonal sprouting after axotomy (Suarez et al. 2006) and to play a prominent role in the development of the nervous system (Nielsen et al. 1998; Waschek et al. 1998). Although PACAP has emerged as a promising candidate for the treatment of autoimmune encephalomyelitis or arthritis (Abad et al. 2001; Kato et al. 2004), further research is needed to clarify the neuronal response to peripheral inflammatory pathology.

In contrast to PACAP, the VIP immunoreactivity in the control DMX was found exclusively in neuronal processes crossing between traced somata. However, aspirin-induced gastritis resulted in de novo synthesis of VIP in nearly 40 % of the gastric vagal neurons. Our finding corresponds with a significant increase in the number of the VIP-IR stomach enteric (as well as spinal dorsal horn) neurons in response to the gastric inflammatory state reported in the rat (Li et al. 2009). Congruently, Sipos et al. (2006) reported on the enhancement of VIP-IR fibers in stomach antrum mucosa during gastritis, which may suggest the participation of VIP in the development of neurogenic inflammation, repairing processes, or chronic gastritis. In contrast, Erin and coworkers (2012) demonstrated a decreased level of VIP in human gastric mucosa during gastritis or ulcers.

VIP, like PACAP, has been reported to be an immunomodulatory and anti-inflammatory factor (Waschek 2013). As an anti-inflammatory molecule, VIP inhibited the production of the pro-inflammatory cytokines TNFα, IL-6, IL-12, and NO as well as stimulated the production of anti-inflammatory cytokine IL-10 in macrophages (Xin and Sriram 1998; Delgado et al. 1999). Moreover, it reduces neutrophil chemotaxis (Sergejeva et al. 2004). It was additionally proven that intraperitoneal injection of VIP prevented stress-induced gastric ulcer formation by inhibiting mast cell degranulation and preventing lipid peroxidation (Tuncel et al. 1998). Due to its anti-inflammatory properties, VIP has been successfully applied as a therapeutic agent in multiple pathologies, such as heart failure, primary pulmonary hypertension, type 2 diabetes, and gastrointestinal motility disorders (Gozes and Furman 2003; Gozes and Furman 2004), and may be a candidate for treatment of various inflammatory processes. Our finding of increased expression of PACAP and VIP in the porcine vagal neurons as a consequence of aspirin-induced gastritis provides convincing evidence of the special role of both peptides in neuronal response to gastric pathology and confirms the pig as a good animal model for a PACAP/VIP anti-inflammatory preclinical study.

Our discovery of de novo synthesis of NOS in approximately 25 % of the FB-positive somata in the ASA group involves nitric oxide (NO) in neuronal response to peripheral inflammatory process. According to former reports, induction of the NOS expression accompanied axotomy (Całka et al. 2004; Gańko and Całka 2013), as well as inflammatory bowel disease (Miampamba and Sharkey 1999) or cystitis (Callsen-Cencic and Mense 1997). Therefore, neurons upregulate NO production in reaction to both peripheral denervation and an inflammatory state of the innervated organ. This increased NOS expression indicates the neuroprotective and anti-inflammatory actions of NO. Moreover, NO synthesis in the stomach constitutes a component of gastric mucosal defense and, consequently, when it is suppressed, the mucosa of the alimentary tract is more susceptible to damage (Whittle 1993). Supporting reports indicate that NO is essential for the maintenance of mucosal blood flow, which is directly responsible for protective mucus secretion (Kubes and Wallace 1995). Moreover, NO prevents leukocyte aggregation (Wallace 1997), while large doses of NO may enhance gut permeability and stimulate apoptosis and intestinal secretion. Additionally, it can decrease inflammation by inhibiting the activation of nuclear factor-kappa B (Dijkstra et al. 2004). However, since NO may exert its anti-inflammatory action through different mechanisms, we still do not know if NO produced and released by vagal gastric neurons exclusively affects the intramural neurons of stomach or due to its high permeability range of up to 300 μm (Całka 2006), it may influence an inflammatory focus directly. This question requires further investigation.

A microscopic analysis conducted in the ASA group revealed the presence of GAL immunoreactivity in 3.4 % of traced perikarya, while in the control DMX (exclusively), a network of GAL-IR fibers was observed between vagal gastric neurons. This finding indicates possible GAL participation in the neural response to inflammatory conditions and is in line with previous reports in the rat (Ji et al. 1995; Calza et al. 1998). Similar upregulation of GAL synthesis has been observed in enteric neurons following formalin-induced porcine colitis (Gonkowski et al. 2010). Inflammatory GAL induction in murine colitis is accompanied by parallel augmentation of GAL1 receptor expression in colonic epithelium (Marrero et al. 2000; Matkowskyj et al. 2009), thus constituting a subsequent link of the GAL signaling pathway. To date, there has been no data specifying the final functional destination of the GAL, although it may affect the secreting neuron itself, postganglionic secondary neuron, or may act on other cells of an inflammatory focus.

Moreover, GAL has been reported to be an important factor affecting neuronal survival (Hobson et al. 2008; Elliott-Hunt et al. 2011). It was revealed that nerve injury upregulates both the GAL as well as its mRNA level in the central nervous system (Cortes et al. 1990). Coherently, peripheral axonal transection of the porcine stomach supplying vagal neurons (Gańko and Całka 2013) induced the expression of GAL in those neurons. In contrast, GAL in cell cultures appeared to inhibit neuronal survival (Arciszewski and Ekblad 2005). Although ASA-induced inflammation evoked de novo expression of GAL in the FB-positive somata, the small number of GAL-IR, compared with PACAP, VIP, and NOS expressing perikarya, may suggest a secondary role of GAL in the neuronal response to the inflammatory state.

In addition to PACAP, VIP, NOS, and GAL immunoreactive vagal perikarya, in the porcine DMX, we observed SP, LENK, CART, GAL, NOS, and VIP immunopositive fibers presenting different density patterns as well as spatial relationship with FB-labeled somata. The SP- and LENK-IR processes formed a dense network whose fibers often ran close to the gastric neurons, therefore suggesting possible functional interaction. Although CART-, GAL-, and NOS-IR fibers presented moderate density and VIP-IR processes were rarely encountered, all of them were found in close proximity to the FB-positive perikarya. This unique spatial relationship between the processes and gastric neurons constitutes the morphological foundation for central regulatory action, indirectly affecting an aspirin-induced inflammatory state. Identification of the SP NK_1_ receptor on vagal efferent somata provides direct confirmation of this pathway (Plata-Salaman et al. 1989; Le Brun et al. 2008). Possible physiological implications of the SP, LENK, CART, GAL, NOS, and VIP immunopositive processes in the regulation of stomach function have been outlined in our former reports (Gańko and Całka 2013; Gańko et al. 2013).

We did not observe changes in density and correlation between SP-IR fibers and FB-positive perikarya between the control group and the ASA group. Our observation disagrees with the report of Sipos et al. (2006), where upregulation of SP immunoreactivity in gastric mucosa during gastritis was observed, indicating involvement of SP in processes related to neurogenic inflammation or chronic gastritis. Therefore, further studies confirming this question are necessary.

Although opioid peptides were found to exert vagally mediated gastroprotective effect on mucosa lesions (Gyires et al. 2001), our research did not reveal an increased expression of LENK in the LENK-IR fibers in ASA DMX. This fact may be a consequence of protective function of other bioactive substances such as PACAP, VIP, NO, and GAL in ASA-supplementation-induced gastritis, but this question needs further research.

In our study, no difference in CART expression between control and ASA DMX was observed. However, previous report proved that chemically induced inflammation affected CART-like immunoreactivity in the descending colon and caused increase of CART-IR fibers and reduction in CART-IR perikarya in myenteric and submucus plexuses (Burliński 2012).

In summary, this study shows that ASA-induced gastritis in immature gilts results in increased expression of PACAP and de novo expression of VIP, NOS, and GAL in dorsal motor vagal neurons supplying the prepyloric region of the porcine stomach. Our findings suggest that vagal gastric neurons respond to peripheral inflammatory changes with increased expression of PACAP, VIP, NOS, and GAL, in consequence, implicating those bioactive substances in neuronal anti-inflammatory response.
